# Fiction and Facts about BCG Imparting Trained Immunity against COVID-19

**DOI:** 10.3390/vaccines10071006

**Published:** 2022-06-23

**Authors:** Gurpreet Kaur, Sanpreet Singh, Sidhanta Nanda, Mohammad Adeel Zafar, Jonaid Ahmad Malik, Mohammad Umar Arshi, Taruna Lamba, Javed Naim Agrewala

**Affiliations:** 1 Immunology Laboratory, Department of Biomedical Engineering, Indian Institute of Technology Ropar, Rupnagar 140001, India; gurpreet.dipas@gmail.com (G.K.); sidhantananda23@gmail.com (S.N.); shaikhadeel143@gmail.com (M.A.Z.); junaidpsst@gmail.com (J.A.M.); md.umararshi@gmail.com (M.U.A.); lamba2414taruna@gmail.com (T.L.); 2 Immunology Laboratory, Division of Cell Biology and Immunology, CSIR-Institute of Microbial Technology, Chandigarh 160036, India; sanpreet2488@gmail.com

**Keywords:** innate immunity, BCG, SARS-CoV-2, COVID-19, vaccines

## Abstract

The Bacille Calmette-Guérin or BCG vaccine, the only vaccine available against *Mycobacterium tuberculosis* can induce a marked Th1 polarization of T-cells, characterized by the antigen-specific secretion of IFN-γ and enhanced antiviral response. A number of studies have supported the concept of protection by non-specific boosting of immunity by BCG and other microbes. BCG is a well-known example of a trained immunity inducer since it imparts ‘non-specific heterologous’ immunity against severe acute respiratory syndrome coronavirus 2 (SARS-CoV-2), the virus responsible for the recent pandemic. SARS-CoV-2 continues to inflict an unabated surge in morbidity and mortality around the world. There is an urgent need to devise and develop alternate strategies to bolster host immunity against the coronavirus disease of 2019 (COVID-19) and its continuously emerging variants. Several vaccines have been developed recently against COVID-19, but the data on their protective efficacy remains doubtful. Therefore, urgent strategies are required to enhance system immunity to adequately defend against newly emerging infections. The concept of trained immunity may play a cardinal role in protection against COVID-19. The ability of trained immunity-based vaccines is to promote heterologous immune responses beyond their specific antigens, which may notably help in defending against an emergency situation such as COVID-19 when the protective ability of vaccines is suspicious. A growing body of evidence points towards the beneficial non-specific boosting of immune responses by BCG or other microbes, which may protect against COVID-19. Clinical trials are underway to consider the efficacy of BCG vaccination against SARS-CoV-2 on healthcare workers and the elderly population. In this review, we will discuss the role of BCG in eliciting trained immunity and the possible limitations and challenges in controlling COVID-19 and future pandemics.

## 1. Introduction

Vaccines provide a long-lived pathogen-specific protective immunity. However, some vaccines, viz., influenza, oral polio, MMR (measles, mumps, rubella), smallpox, measles, BCG, etc., can also provide non-specific cross-protection against other pathogens. The non-specific cross-protection against unrelated diseases has been described for other vaccines such as influenza, oral poliovirus, smallpox, and measles vaccines. These heterologous effects emerge from vaccine-induced immunomodulation. Various studies have shown non-specific protective effects after immunization with an unrelated vaccine or microbial antigens (Table 1). This *de facto* immunological memory occurs in innate immune cells and has been termed ‘trained immunity’. A deeper understanding of the mechanism of trained immunity-based vaccines may result in the next generation of broad-spectrum vaccines and can be a viable approach to fighting COVID-19. The innate immune system is composed of monocytes, macrophages, neutrophils, and NK cells, which respond rapidly and non-specifically upon encountering pathogens. Innate immune cells have the capacity to display an enhanced and robust immune response upon reinfection, which is associated with an increased antimicrobial function of innate immune cells (IICs) [1].

BCG is the only available vaccine against *Mycobacterium tuberculosis (Mtb).* It is a live and attenuated strain of *Mycobacterium bovis.* In addition to its protective role against *Mtb,* BCG vaccination provides non-specific cross-protection against other unrelated pathogens [16,17]. BCG has been shown to provide protection against non-mycobacterial infection in mice lacking functional B-cells and T-cells (SCID mice). BCG vaccination in healthy individuals induces epigenetic reprogramming of monocytes, providing protection against unrelated pathogens for up to 3 months after vaccination [18]. Consequently, this demonstrates the ability of innate immune cells (IICs) to mount a non-specific ‘memory-like’ response [19,20]. A 60-year follow-up of a clinical trial reported a significant reduction in the development of lung cancer in adults that received early childhood BCG vaccination [21]. Additionally, BCG vaccination can alter the phenotype of monocytes that secrete a higher amount of cytokines, such as TNF-α, IFN-γ, and IL-1, upon exposure to non-mycobacterial pathogens [1]. Immunization with the BCG vaccine protects against various viruses, such as the vaccinia virus and herpes simplex virus type 2 [22]. The protective effects of BCG have been shown against influenza A in reducing viral titers, along with reduced inflammation and lung injury [3,22]. A significant increase in non-specific antibodies, enhanced seroconversion against the H1N1 virus, and the augmented phagocytosis of airborne pathogens and memory T-cells in the lungs [23,24] suggest the protective role of the BCG vaccine against unrelated pathogens. Boosting innate immune responses by BCG to control viral replication in the early phase of infection may be a good approach. Importantly, the memory characteristics of trained immunity are distinctly different from adaptive immunological memory as it involves non-specific changes in the number and/or function of IICs, which result in increased resistance to a broad range of secondary infections [25].

It is imperative to precisely understand the structure of pathogens and the immunogenic components for the development of novel and successful vaccines. However, the development of a new vaccine and clinical trials for evaluating its safety and efficacy take considerable time. During SARS-CoV-2 infection, deregulation of innate immune responses results in systemic inflammation, cytokine storms, mass virus replication, highly infectious patients, and chronic forms of the disease. Imparting trained immunity to mount an early response may represent a promising strategy to control COVID-19-like pandemics. Training of immune and non-immune cells can lead to efficient local innate immunity and the elimination of pathogens, curtailing the spread of diseases [26]. There is a more rapid and stronger innate immune response during the trained state against secondary infections [2,27,28]. The induction of trained immunity in monocytes, macrophages, dendritic cells, T-cells, and NK cells includes mechanisms such as histone modification, chromatin remodeling, methylation reprogramming, and metabolic changes. The chromatin modifications induced by BCG vaccination reprogram bone marrow progenitors and stimulate myelopoiesis. These modifications lead to the generation of trained immune phenotypes in cells, with a greater capacity to defend. BCG-mediated innate immune memory is known to be mediated by monocytes. However, the myeloid cells remain in circulation for a limited span, and the mechanism behind the long-lasting memory function of myeloid cells, lasting from several months to years after the initial vaccination, remains undefined [2,18]. BCG is already known for its protective role against pulmonary infections such as TB and H1N1. Therefore, non-specific protective effects elicited by BCG via trained immunity are conjectured to offer protection against SARS-CoV-2 as well [27,29,30]. The results published on the protective efficacy of the vaccines developed against SARS-CoV-2 remain doubtful. BCG has a strong potency of initiating trained immunity in hosts through immunomodulation, the sharing of immunodominant epitopes with the pathogens, and the rewiring of epigenetic, metabolic, and functional machinery. BCG bolsters the immune response by not only training innate immune cells but also through the activation of heterologous T-cells. Although it is difficult to currently predict the protective efficacy of BCG towards SARS-CoV-2, the data suggest that people vaccinated with BCG and living in TB-endemic zones are better protected against the disease than those residing in TB non-endemic sectors. Thus, the elicitation of trained immunity by BCG and non-tuberculous mycobacteria (NTM) might reduce the spread of the infection and represent an important strategy to check the rapidly spreading disease [31].

Presently, several clinical trials and studies are in progress, elucidating the role of BCG vaccination against COVID-19. The population not vaccinated with BCG will be devoid of trained immunity and, therefore, may lack cross-protection against COVID-19, indicated in the protection against disparate viruses and lower overall mortality. Due to the very high mutation and mortality rates caused by SARS-CoV-2, the development of a 100% efficacious vaccine may take longer, and, in this scenario, even transient protection against the virus would be very valuable to control the high transmission and mortality rate of COVID-19. Hence, elucidating the mechanism of BCG vaccination response against SARS-CoV-2 may be an interesting area to explore for the development of an efficient vaccine. Importantly, substantial research is required before concluding that the immunity elicited by BCG can be an essential strategy for COVID-19-like pandemics.

## 2. Induction of the activation of Trained Immunity

**Figure 1:**
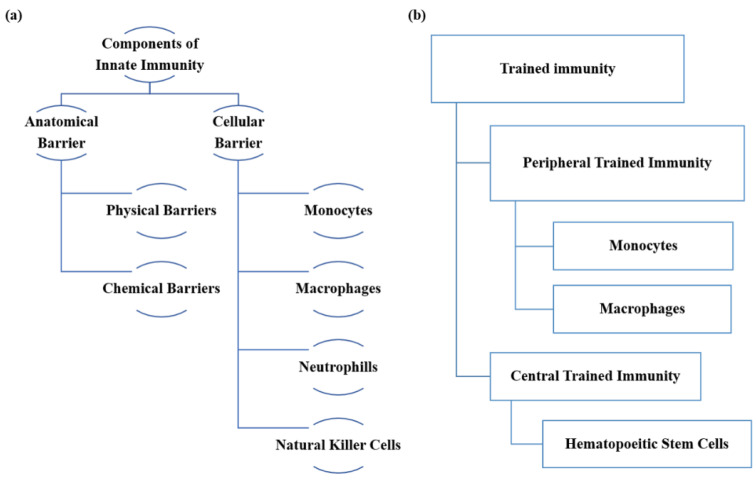
The components of innate immunity and trained immunity. (**a**) Schematic representation of different components of innate immunity. The innate immune system is divided into various subsets to generate the first line of defense against the array of invading pathogens. (**b**) Different cells of trained immunity are responsible for definitive functions.

Innate immunity is the first line of defense against any infection (Figure 1a,b). Both myeloid (neutrophils, monocytes, macrophages) and lymphoid cells (NK cells, B-cells, and T-cells) are major cells that help in the activation of the host defense to protect from various infections. Tight junctions and mucus secretion by epithelial cells initially hinder the entrance of pathogens. Further, innate cells interact with infectious agents through their pathogen-associated molecular patterns (PAMPs) and release soluble mediators in the form of complements, cytokines, chemokines, and reactive free radical species, which ultimately destroy the pathogens. These innate cells (macrophages and dendritic cells) constitute the mononuclear phagocyte system and bolster innate immunity against pathogens [32].

BCG vaccination helps in the clearance of early mycobacterial infections through the induction of trained immunity. It can control non-specific infections such as malaria, *S. aureus*, *C. albicans,* Leishmania and other infections [33,34,35,36]. Further, herpesvirus infection has been shown to protect against various bacterial infections such as *Listeria monocytogenes* and *Yersinia pestis,* indicating the generation of protective trained immunity [37]. Training of human monocytes in vitro with β-glucan shows an improved response against *C. albicans,* which amplifies further upon secondary stimulation with TLRs [38]. All these findings illustrate the manifestation of trained immunity. The concept of trained immunity and its mechanism of action is now considered for developing trained immunity-based vaccines (TIbVs) as an alternative to conventional vaccines. Trained immunity involves the epigenetic modifications and reprogramming of various transcriptional and metabolic pathways in response to endogenous and exogenous stimulation. The epigenetic changes alter the path of downstream signaling. In innate immune cells, the continuation of this epigenetic transformation even after the removal of stimulus is known as immunological memory, i.e., the ‘training’ of the cells, which results in a faster and more robust immune response even in the presence of lower concentrations of antigens to related/unrelated pathogens [25].

**Figure 2:**
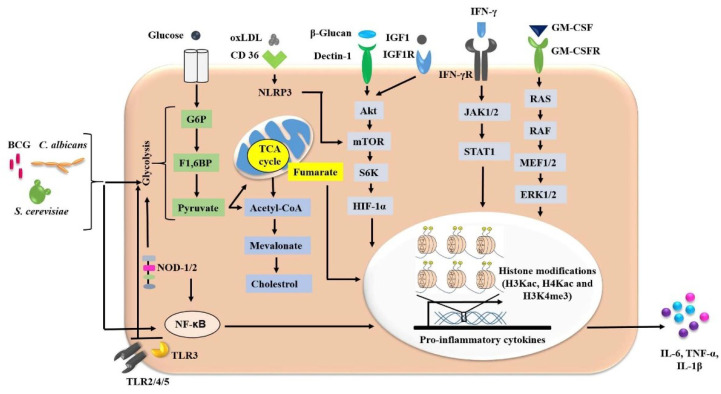
The signaling pathways that operate in trained immunity. The exposure of innate immune cells to various stimuli initiates the trained immunity pathways. Interaction or exposure with microorganisms (BCG, *C. albicans, S. cerevisiae*), endogenous/soluble stimuli (GM-CSF, IFN-γ, β-glucan, oxLDL, IGF1, lipoproteins), or PAMPs with surface/cytosolic receptors leads to metabolic shifts and epigenetic modifications in these innate immune cells. This initiates a series of signaling cascades and increases the secretion of pro-inflammatory cytokines. Intermediates of these signaling pathways (Akt-mTOR-HIF-1α, JAK/STAT, RAS, NF-κB) regulate the genetic machinery through acetylation and methylation processes. Activation of glycolysis and deposition of fumarate (TCA cycle) and mevalonate (cholesterol synthesis) play important roles in the induction of trained immunity. TLR (Toll-like receptor), NOD (nucleotide-binding oligomerization domain), oxLDL (oxidized low density lipoprotein), IGF1 (insulin-like growth factor1), IGF1R (insulin-like growth factor1 receptor), mTOR (mammalian target of rapamycin), JAK (Janus kinase), STAT (signal transducer and activator of transcription), GM-CSF (granulocyte monocyte-colony stimulating factor), IL (interleukin), TNF-α (tumor necrosis factor-α), HIF-1α (hypoxia inducing factor-1α), G6P (glucose 6-phosphate), F1,6-BP (fructose 1, 6 bisphosphate), TCA (tricarboxylic acid).

Long-term responses associated with trained immunity are based on the reprogramming of myeloid cells by stable epigenetic changes. Epigenetic or metabolic reprogramming in monocytes and macrophages depends on various stimuli and key signatures for the stimulation of trained immunity [39,40] (Figure 2). Upon exposure to β-glucan, the α dectin-1-dependent AKT/mTOR/HIF-1α signaling pathway was induced in innate immune cells, making these cells more responsive to secondary attack [41].

### 2.1. Trained-Immunity-Based Vaccines and Associated Mechanism

**Figure 3:**
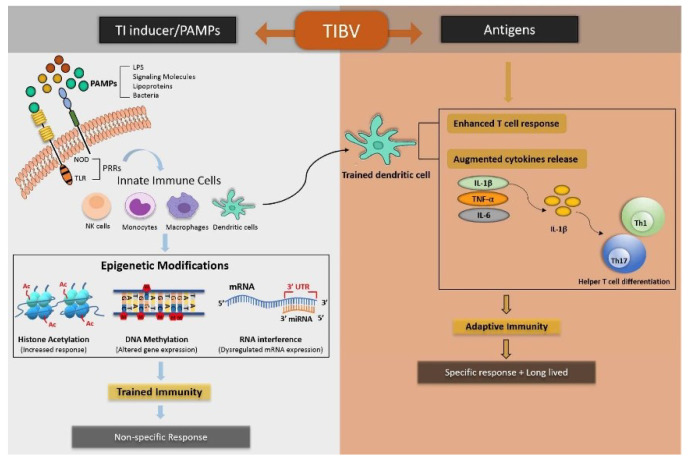
Mechanism of trained immunity-based vaccines. TibVs can induce both non-specific and specific immunological memory against heterologous pathogens. Non-specific immune memory is generated by trained immunity through epigenetic modifications in IICs in response to pathogen-associated molecular patterns (PAMPs). On the contrary, specific adaptive immune response/memory is produced against nominal antigens carried by TibVs through antigen presentation by APCs (antigen-presenting cells) as well as by the release of pro-inflammatory cytokines such as IL-1β, IL-6, and TNF-α, secreted by the trained IICs.

The broad spectrum of protection imparted by trained-immunity-based vaccines (TIbVs) is achieved by either activating non-specific IICs, viz., monocytes, macrophages, and NK cells, or by maintaining DCs in an activated state to drive T-cell adaptive immune response (AIR) against specific and related bystander antigens [20]. The design of a TIbV requires a suitable PRR (pattern recognition receptor) ligand from a targeted pathogen that is considered essential for trained immunity as well as AIR. The chosen PRR ligand plays a decisive role in the success of TIbVs. The whole organism or its derived products should consist of specific pathogen-associated molecular pattern molecules (PAMPs) of trained immunity inducers, along with specific antigens against which AIR is aimed. The second most important characteristic of TIbVs is that they should not only trigger an immune response against a nominal antigen but also a heterologous antigen. As a result, TIbVs can divulge wide-spectrum and continuous resistance against heterologous infection (Figure 3). The proper and accurate targeting of IICs may result in a specific and non-specific immune response. Trained IICs such as DCs and macrophages can stimulate AIR against nominal antigens used in the TIbV [42]. Further, AIR is amplified by the upregulation of the expression of PRRs in IICs [18,43]. As a consequence, TIbVs can instruct both specific and non-specific immunity against nominal and unrelated pathogens. Furthermore, conventional vaccines have shown their effect on trained immunity, which is evident by the fact that the live vaccinia virus smallpox vaccine can not only protect against smallpox but also measles, scarlet fever, and whooping cough [44,45,46].

Different mechanisms are involved in the induction of trained immune responses during infections. BCG vaccination in healthy humans induces the transcriptional changes in the progenitor population of HSCs and hence better protection against the heterologous infection. It has been shown that BCG changes the transcriptional profile of HSCs in bone marrow and educates them to generate epigenetically modified monocytes/macrophages that provide far better protection against *Mtb* compared to naïve macrophages. Modulating HSCs in the bone marrow can be a novel strategy to counter the infection through induction of trained immunity [47]. In the absence of AIR, Thy1^+^ NK cell-dependent memory can protect from subsequent infections from the vaccinia virus [48]. The BCG remedial effect against bladder cancer was an impact of trained immunity [49,50]. BCG interacts within a NOD-2-dependent pathway and triggers the production of monocyte-derived cytokines TNF-α and IL-6 in response to *in vitro C. albicans*, *S. aureus,* and *Mtb* [2,38]. The live attenuated influenza vaccine protects children against respiratory syncytial virus (RSV) infection through the induction of trained immunity [51,52]. Further, it can modulate the function of monocytes and macrophages by induction of the enhancement of the expression of costimulatory molecules and PRRs, such as TLR-4. Consequently, it expands the host’s defense against pathogens [53]. Besides functional and epigenetic mechanisms, metabolic processes leading to selective accumulation or depletion of certain metabolites of the central metabolism regulate trained immune responses. They function as co-regulatory molecules for epigenetic enzymes [54].

### 2.2. Clinical Applications of TIbVs

TIbVs have been used for clinical purposes since the 1900s. They defend us from pathogens that cause recurrent infections. During the influenza pandemic, bacterial vaccines were used to protect against secondary infections of *S. pneumoniae*. Intriguingly, it also enhanced immunity against the influenza virus [55]. The yellow fever vaccine provides non-specific immunity by triggering the long-term activation of monocytes and NK cells [56]. Sublingual vaccine MV130 is used to treat frequent respiratory and urinary tract infections [57,58]. The vaccinia vaccine elicits both adaptive immune response (AIR) and innate immune response (IIR). It induces IIR via TLR-2 signaling in macrophages and protects against melanomas [56,59]. The Marek’s disease vaccine induces protection by trained immunity by activating macrophages and increasing the secretion of IFN-γ, IL-1β, IL-8, IL-12, and TNF-α in domestic chickens [60]. These cytokines regulate NK cells and macrophage activation, IFN-γ production, and Th1 immunity. Four population-based cohort studies were done to study the possible effects of measles-containing vaccine regimens in high-income countries. They found that the MMR vaccine has a particularly beneficial effect on respiratory infections [61,62,63,64]. Measles-based vaccines were helpful in greatly reducing child mortality in low-income communities in Haiti [65]. In 2011, a Finnish trial reported a protective effect against acute otitis media from oral polio vaccines (OPVs) administered to children in the age group of 6–8 months [66]. Similarly, a randomized controlled trial in Bangladesh showed a significant shortening of the length of diarrhea periods in OPV-vaccinated children [67]. They also protect against mucosal infections such as pneumonia and wound and urinary tract infections in young and old people and individuals with suppressed immunity [68]. Further, certain adjuvants and immuno-modulators can enhance IIR by evoking trained immunity. These vaccines can boost immunity in clinical conditions and diseases related to immune paralysis and sepsis [69]. Moreover, the epigenetic and transcriptional regulation of IICs is critical for understanding the induction of trained immunity against infectious diseases [39,41].

### 2.3. Role of Trained Immunity in Viral Diseases

The induction of trained immunity by NK cells, macrophages, neutrophils, mast cells, eosinophils, basophils, and ILCs is important for the efficient control of viral infections [70]. There are multiple mechanisms through which IIR exerts its antiviral effects. NK cells are the first line of defense against tumors and viral infections. The activation of NK cells is achieved through a combination of signals that involves several inhibitory and activating receptors, many of which engage MHC class I-like or class II-like proteins as their ligands [71]. The absence of MHC class I on target cells often leads to NK cell activation. The phagocytosis of opsonized and non-opsonized viruses takes place by macrophages, DCs, and neutrophils. Recently, MV130, a bacterial vaccine against recurring respiratory tract infection, was reported to generate a T-cell response against unrelated flu antigens and showed protective efficacy against respiratory tract viral infection via induction of trained immunity [58,72]. Vaccination with BCG may provide better protection against viral infections [73]. BCG vaccination of healthy volunteers showed an increase in the secretion of pro-inflammatory cytokines after ex vivo stimulation of NK cells with mycobacteria and other unrelated pathogens, which persisted for three months post-vaccination. NK cells may contribute to the non-specific (heterologous) beneficial effects of BCG. Vaccination with BCG led to better protection in SCID mice, which was partially dependent on NK cells [74]. Studies have shown that NK cells can display long-lived memory and contribute to secondary immune responses [75,76]. Antibody-dependent cellular cytotoxicity (ADCC) is mediated by NK cells, mast cells, basophils, and eosinophils. Further, the ILCs, DCs, and macrophages present the viral antigens in context with MHC molecules to the adaptive immunity cells (AICs) [70,71].

A gamma herpesvirus infection could protect against subsequent allergic asthma by modulating lung innate immune cells in mice [77]. The induction of long-term memory alveolar macrophages during adenovirus infection was found to be critical for trained immunity, which provided increased protection against bacterial infection [78]. These studies suggest that trained immunity can be induced in specific organs, such as lungs, which can be beneficial in infections such as SARS-CoV-2 [22]. A clinical trial conducted in Greece in 2017 concluded a 53% decrease in new infections and an 80% decrease in common respiratory tract infections in the BCG-vaccinated group (NCT03296423). A much deeper understanding is required to design more effective and safer vaccination strategies against viral infections.

### 2.4. Immune Response to SARS-CoV-2

The SARS-CoV-2 virus causing COVID-19 was reported in December 2019 in Wuhan in the Hubei province of China. It is a respiratory disease-causing pneumonia-like symptoms in severe cases, with bilateral diffuse alveolar damage, pulmonary edema, acute respiratory distress syndrome (ARDS), and characteristic syncytial cells in the alveolar lumen, affecting other vital organs of the host [79]. Angiotensin-converting enzyme 2 (ACE-2) is the main receptor of SARS-CoV-2 on the membrane of target cells [80,81]. The binding of ACE-2 and the receptor-binding domain (RBD) induces a conformational change in the ‘S protein’, which leads to the cleavage of S1 and S2, mediated by serine protease TMPRSS2. This facilitates the fusion of virus envelopes with the cell membrane by the S2 protein, thus allowing the viral RNA to enter the cytoplasm of the target cell [82,83,84]. SARS-CoV-2 infection incites autophagy, inhibits ACE-2 expression, and induces basal membrane detachment [85,86]. Therefore, it leads to the binding of the AT1aR receptor to angiotensin II and causes acute lung damage [87].

Many recent studies have elucidated the role of SARS-CoV-2 in the stimulation of IICs and the activation of AIR. It has been reported that SARS-CoV and SARS-CoV-2 equally infect alveolar macrophages and type-I and type-II pneumocytes. SARS-CoV-2 triggers lower levels of IFNs and pro-inflammatory cytokines/chemokines compared to the SARS-CoV strain in spite of higher viral loads in human lung tissues [88]. Upon comparative transcriptional analysis of major viral pathogens, it was observed that SARS-CoV-2 reduces IFN-I and IFN-II responses, whereas it significantly augments the yields of IL-1β, IL-6, TNF-α, and IL1RA. This study was further supported by the increased serum levels of these molecules in COVID-19 patients [89]. SARS-CoV-2 increases the production of pro-inflammatory cytokines IL-6, MCP1, G-CSF, MIP1A, TNF-α, and GM-CSF [90]. Epithelial cells play a major role during SARS-CoV-2 infection. Infected lung epithelial cells produce IL-8, which recruits neutrophils and T-cells. SARS-CoV and SARS-CoV-2 instigate an inflammatory reaction and the activation of the inflammasome and IL-1β pathway via the activation of TLR-3 and TLR-4 [91].

The shift between IIR and AIR is critical for the clinical progress of SARS-CoV-2 infection. T-cells, B-cells, macrophages, and DCs do not express ACE-2 receptors. However, some studies suggest that the presence of DC-SIGN on DCs may act as a trans-receptor for SARS-CoV-2 and transfer the virus to other cells [92]. CD26 is an activation marker present on the surface of T-cells and NK cells. It binds to the S1 protein of SARS-CoV-2 and plays a key immunoregulatory function during viral infections [93,94]. During SARS-CoV-2 infection, elevated glucose levels may impair T-cell function and lead to lymphopenia [95]. Severely infected patients with SARS-CoV-2 exhibit a significant reduction in the total number of T-cells [96,97]. Harnessing innate immunity to potentially fight SARS-CoV-2 might be a novel strategy, wherein immunomodulation through TLR/NLR agonists may provide a plausible solution [98].

### 2.5. BCG and Trained Immunity against COVID-19

BCG is a prominent example of trained-immunity-inducing vaccines, which can be explored to overcome the problem associated with COVID-19 disease control. BCG is one of the prime examples of vaccines that have trained immunity effects. BCG-induced trained immunity bolsters the function of innate immune cells, as proven by the improved release of cytokines and reactive oxygen species upon secondary stimulation with non-related pathogens. Studies have suggested that the non-specific protection instilled by BCG is not primarily mediated by changes in innate immune cells but by the lymphocyte-driven mechanism. Epigenetic control of pro-inflammatory cytokine gene expression by BCG is mainly dependent on the NOD2 signaling in monocytes [2,23]. Through trained immunity, BCG can stimulate skin DCs to secrete IL-6, IL-12, and TNF-α and stimulate both CD4 T-cells and CD8 T-cells [44,45]. Further, it can modulate the function of monocytes and macrophages by induction of the enhancement of the expression of PRRs. Consequently, it expands the host’s defense against pathogens [53]. Trained immunity has also been induced in myeloid progenitor cells of the bone marrow, resulting in the production of monocytes with heightened immune potential for a longer duration [47]. Scientists all over the world are divided over the view that BCG vaccination may prove to be a reasonable solution against SARS-CoV-2 until an effective vaccine is developed. The low dose of BCG, inoculated through the intranasal route, offered protection against the influenza A/Puerto Rico/8/34 (PR8) (H1N1) virus by inducing efferocytosis of alveolar macrophages [22]. Thus, priming innate immunity could result in a faster and more robust immune response against a broad spectrum of pathogens, including viral, bacterial, and fungal infections. The protective effect of the BCG vaccine against *Mtb* is not lasting long and decreases with time. Therefore, making a precise estimate for BCG efficacy is very difficult and needs extensive validation before reaching a concrete conclusion [99,100,101].

**Figure 4:**
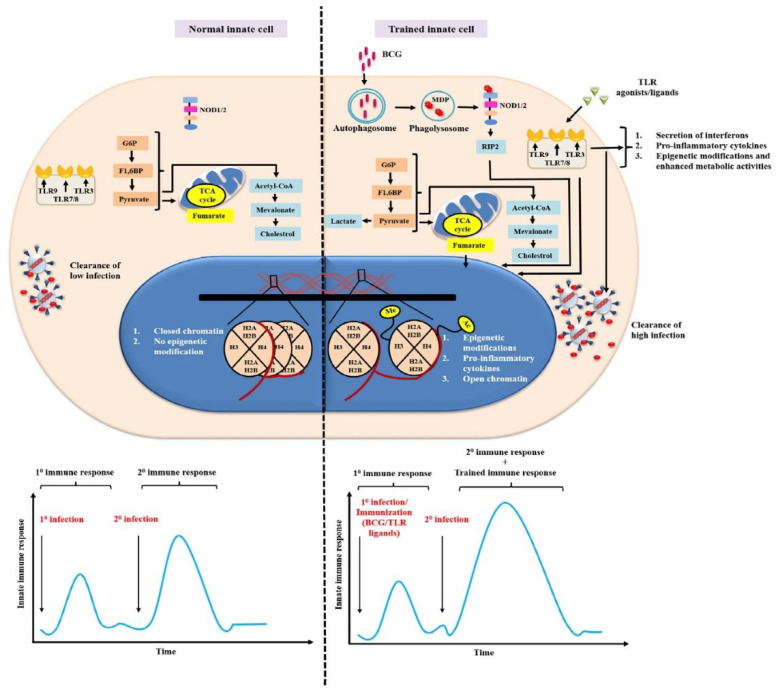
Triggering trained immunity to enhance the immune response. Innate memory can be created by training IICs with vaccines or immunomodulators. The trained cells revert faster during reinfection with the same or unrelated pathogens. In an untrained innate immune cell, low infection is easily cleared off through the secretion of pro-inflammatory cytokines and other soluble mediators. In the case of microbial ligands and BCG-trained cells, reprogramming of epigenetic and metabolic machinery takes place, leading to an enhanced innate immune response. The chromatin structure opens up, leading to the binding of the transcription factors and enhancers, ultimately increasing the cell’s responsiveness to various pathogens.

As per the Center for Systems Science and Engineering report (CSSE, Johns Hopkins University), US, Italy, and Spain showed the highest contagion rates, with a sustained increase after the first reported cases. The Netherlands and Germany also showed a significant increase in their confirmed cases per million inhabitants [102]. The BCG vaccine has been shown to induce heterologous lymphocyte responses against non-specific antigens by eliciting higher production of memory CD4 T-cells and CD8 T-cells to generate an effective immune response against SARS-CoV-2 [103,104,105]. Recently, it was shown that immunization with wild-type or recombinant BCG (expressing viral antigens) resulted in an enhancement of a non-specific immune response, with increased secretion of pro-inflammatory cytokines and T-cell activation upon antigenic stimulation [106]. Vaccination with BCG promoted a trained immunity profile in the immunized mice, with increased secretions of IL-6 and TNF-α, thereby contemplating a possible reality of BCG vaccines in the immune defense against non-related respiratory diseases [73] (Figure 4).

In moderate cases of SARS-CoV-2 infection, trained-immunity-induced responses via BCG can offer prophylactic protection. This results in the controlled production of pro-inflammatory cytokines such as IL-1β, TNF-α, and IFN-γ by ‘trained’ innate immune cells, resulting in the quicker removal of the virus, decreased viremia, and restricted inflammation. IFN-γ can further promote B-cell activation and the early production of neutralizing IgG antibodies, facilitating faster removal of viruses.

Training of immune and non-immune cells can lead to efficient local innate immunity and the destruction and removal of the virus before it causes disease or spreads. However, since BCG is a live vaccine, patients suffering from immune-mediated inflammatory diseases, HIV patients, or people on immunosuppressive drugs should be excluded from BCG vaccination as they are at a much higher risk for severe complications or organ failure. Therefore, the use of the BCG vaccine may be a limited source of protection in a limited population. During SARS-CoV-2 infection, there is less production of type-1 interferons such as IFN-α initially when the viral load is on the rise [89]. Hence, the virus gets an opportunity to evade primary immune surveillance. BCG vaccination can help in antiviral responses by reinforcing innate immunity, which helps in the restoration of type-1 IFN signaling. Various randomized clinical trials using BCG have started to determine its protective efficacy against COVID-19. Interim analysis of the phase III ACTIVATE trial was conducted to evaluate the protective efficacy of BCG in elderly patients against respiratory infections (NCT03296423). The results of the study revealed the safety and protective efficacy of BCG vaccination in the aged population against viral respiratory infections [107]. A study performed on children could not demonstrate the non-specific effect of BCG in reducing the number of infectious illnesses from birth to 15 months [108]. Furthermore, it did not protect against parent-reported infections in infants of less than 13 months [109]. These studies also call for further studies of the role of the BCG strain, time of administration, and route of delivery in the non-specific effects of vaccines. A Phase III clinical trial is in progress to test the efficacy of the BCG vaccine in strengthening innate immunity against SARS-CoV-2 infection in frontline health workers (NCT04384549). Several trials are ongoing in Europe and Australia to evaluate the efficacy of BCG vaccination in high-risk and old populations (Table 2). Another trial for reducing the susceptibility of disease in health care workers by BCG vaccine against SARS-CoV-2 is known as BCG-CORONA (NCT04328441).

BRACE is a two-group, multicentric phase III randomized controlled trial involving BCG vaccination to protect healthcare workers against COVID-19 (NCT04327206). In this study, healthcare workers receiving a single dose of the BCG vaccine will be monitored for 12 months. Another trial is known as ‘BCG as Defense against COVID-19 (BADAS)’ (NCT04348370). These trials are based on the assumption that vaccination with BCG in healthcare workers may reduce the severity and occurrence of COVID-19; this assumption is based on a preliminary report showing that areas with existing BCG vaccination programs have a low incidence of COVID-19. In contrast, a small study conducted in Israel involving BCG-vaccinated and non-vaccinated groups demonstrated no difference in the susceptibility to SARS-CoV-2 [110]. These studies prove that BCG vaccination does not induce life-long protection. This also indicates the induction of a relatively limited duration of trained immunity post-BCG administration. The results from these ongoing trials will decipher the protective efficacy of BCG against SARS-CoV-2 [111] and possibly help to elucidate the mechanism of protection offered by the vaccine. Though they remain unclear right now, the outcomes of the clinical trials in progress may give us an idea about the heterologous immune responses and protective mechanisms induced by trained immunity via BCG vaccination. However, several confounding factors, such as geographical features, age differences, diagnostic testing rates, type and mode of data collection, regulatory controls, and public behavior, may affect the accuracy of these trials. Interestingly, BCG elicits an immune response by training not only IICs but also heterologous T-cells. BCG vaccination augmented the non-specific immune response of both innate trained immunity and heterologous Th1/Th17 responses that were sustained for one year post-vaccination [18]. It is worth mentioning here that provoking trained immunity may inflict a lethal repercussion due to the extensive release of cytokines. Excessive production of cytokines, termed a ‘cytokine storm’, has been reported in COVID-19 patients. However, this can be controlled by judiciously optimizing the dose and duration of BCG inoculation. Without the controlled and definite results of the clinical trials, it is too early to predict the outcomes related to BCG vaccination [112]. Furthermore, as already proven, the protective efficacy of BCG against childhood forms of TB may last for several years but the heterogenous non-specific protection is likely much shorter. The duration of non-specific innate immune memory in mice lasts for at least 3 months [74], whereas epidemiological data showed that unspecific protective effects might last 3–5 years in humans [113]. This indicates that the trained immunity approach may not be a permanent solution and could serve as a temporary option in controlling COVID-19 morbidity and mortality. Therefore, the development of a specific, well-defined vaccine is of utmost importance.

Efforts have also been initiated to test other TB vaccine candidates for their protective efficacy against COVID-19. A joint Germany–India project has been implemented to check the efficacy of a genetically modified BCG vaccine that expresses listeriolysin (Hly) of *Listeria monocytogenes* (VPM1002) for its probable protection against TB and COVID-19 in health workers and the elderly population [114].

Besides the advantages of using innate memory induction by BCG, there are certain challenges before using BCG as a potential vaccine in mass vaccination for COVID-19. Being the only vaccine in use for TB, the original strain has not been cloned or preserved but is merely sub-cultured in different settings. The manufacturing conditions of vaccines also play an important role in deciding the efficacy of the vaccine. BCG vaccine production faces difficulties in GMP issues, quality, outdated technology and products, and licensing [115]. Therefore, there is a strong difference seen in the immunological and microbiological properties of different strains. Hence, it is important to identify the best strain and manufacturing conditions since a change on a small scale may also amount to significant variations in the population. Further, the dose and route of administration also play an important part. Though the BCG vaccine is administered intradermally, there are reports of varied effects of different routes of vaccination on immunological responses. Intranasal or endobronchial administration in non-human primates induced much more effective protection than any other route [116]. Recently, a study reported that the intravenous administration of BCG substantially limited *Mtb* infection in nine out of ten rhesus macaques [117]. Pulmonary mucosal immunization was shown to be more efficient than intradermal administration against TB [118]. Since SARS-CoV-2 infects through the respiratory route, intranasal vaccination could be an advantage for the induction of trained immune responses in the host. Adept training of epithelial cells in normal healthy individuals may lead to an efficient immune response to fight COVID-19. Hence, the route of vaccination and the dose have profound implications for vaccine delivery and clinical development. Most importantly, BCG is also routinely administered in the mass vaccination of infants and children. If this vaccine is to be considered against SARS-CoV-2, stringent measures have to be in place so as to ensure the availability of a vaccine, especially in low- and middle-income countries, and they must not adversely affect routine infant vaccination. Additionally, it is very important to first analyze the effect of the BCG vaccine on patients suffering from other complications such as diabetes, autoimmune diseases, and cardiovascular diseases because this category of patients is at even higher risk of COVID-19; their non-specific immune response may pose the threat of adverse reactions. Hence, it is not advisable, as of now, to administer preventive BCG vaccines to patients with any form of compromised immune system. The severity of side effects following BCG vaccination may differ depending on the strain used, the dose of the vaccine, and the immunological and health status of patients. Therefore, it is necessary to monitor the patients and maintain regular follow-ups in case of any adverse events.

## 3. Summary

Vaccines provide everlasting protection by eliciting adaptive immunity and generating long-term immunological memory. However, some vaccines evoke non-specific, heterologous, and cross-protection against related pathogens such as smallpox and poliovirus. Several studies have reported non-specific protective effects against infections after immunization with an unrelated vaccine or microbial antigens. This could be a viable approach to fighting pandemics such as COVID-19. BCG vaccination leads to the metabolic, epigenetic, and functional reprogramming of innate cells, leading to enhanced protection against secondary infections. BCG vaccination bolsters the secretion of pro-inflammatory cytokines in healthy individuals. Additionally, many studies have shown protective efficacy against various viral infections, such as influenza, respiratory syncytial virus, human papillomavirus, and herpes simplex virus [73]. Several clinical studies have also reported that BCG can offer protection against lower and upper respiratory infections. BCG improves the human body’s immune response, involving antigen-specific T-cells and memory cells. It also induces adaptive functional reprogramming of mononuclear phagocytes that generate protective effects against different respiratory infections [119]. Basically, COVID-19 is a respiratory disease that causes pneumonia-like symptoms in severe cases, with bilateral diffuse alveolar damage, pulmonary edema, acute respiratory distress syndrome, and characteristic syncytial cells in the alveolar lumen, affecting other vital organs of the host [74]. The SARS-CoV-2 infection leads to dysregulation of the innate immune response, which results in systemic inflammation, high cytokine storms, and high virus replication in infected patients with chronic forms of the disease.

A trained-immunity-based vaccine for respiratory or other mucosal infections could produce a sterilizing immunity that prevents the development of an active infection or the establishment of latent colonization. This could lead to a better adaptive response or an increased innate immune response. The BCG vaccine is a promising candidate molecule against viral infections such as SARS-CoV-2 and Andes orthohantavirus, inducing a marked antigen-specific immune response [106]. Presently, there are no reliable or perfect options available for the treatment of COVID-19, as evinced by the incessant mortality and morbidity globally. Further, the doubtful results of the available vaccines to prevent infection set a challenge for the continuing search for dependable and effective treatment for SARS-CoV-2 [118,120,121,122]. A vaccine that can induce protection and immunity against the array of SARS-CoV-2 variants is the best resort to stop the current pandemic. Since the virus has very high transmission and mortality rates, even temporal protection would be very beneficial at any stage. As BCG is already known for its protective efficacy against various viral and non-viral diseases, it may serve as an important alternative strategy to bridge the period before an efficient vaccine is available. Most importantly, induction of non-specific immune responses through trained immunity can be a novel option to improve antiviral defense against SARS-CoV-2 infection [16,23,26].

Trained-immunity-based vaccines (TIbVs) are developed on the concept of inducing trained immunity through (i) the revamping of the epigenetic machinery of innate immune cells, (ii) immuno-modulating innate immunity through PRR–PAMP interactions, (iii) sharing homologous epitopes with the pathogens. Ultimately, trained immunity can be a potential alternative to decrease dissemination and mortality until suitable vaccines against pandemics are available. However, the results of ongoing clinical trials need to be ascertained before arriving at any conclusions regarding the BCG vaccine.

## 4. Expert Opinion

Only vaccines generating long-lasting protective immunity can eliminate the COVID-19 disease. Many vaccines with uncertain protection are now available against COVID-19; however, no concrete data is available on the persistence of immunological memory. All these vaccines have been derived from either inactivated whole virus or its components, eliciting B-cell and T-cell responses.

People living in India and many Asian and African countries are BCG-vaccinated. Furthermore, these are TB-endemic regions with a high burden of latent TB [31,123]. The data suggest that the inhabitants of these countries showed less morbidity and mortality compared to the populations living in countries that have stopped BCG vaccination. The protection in BCG-vaccinated subjects may be due to the presence of trained immunity against COVID-19.

It is well known that BCG has a strong propensity to modulate immunity through trained immunity. It is also intriguing to suggest that trained immunity can be harnessed for preventive and therapeutic purposes. BCG may prove to be a potential TIbV against SARS-CoV-2. There is a need to characterize the innate immune activation by SARS-CoV-2 as well as the influence of the BCG vaccine in inducing inflammatory responses. A recent study demonstrated the efficacy of BCG in combination with a stabilized, trimeric form of the SARS-CoV-2 spike antigen (BCG:CoVac). The study showed rapid development of virus-specific IgG antibodies, along with high titers of SARS-CoV-2 neutralizing antibodies and T-cell responses in animals. The BCG:CoVac vaccine effectively neutralized B.1.1.7 and B.1.351 SARS-CoV-2 variants of concern. This study highlights the ability of BCG-based vaccines to protect against major SARS-CoV-2 variants circulating globally. Although the mechanisms of BCG-induced immunomodulatory effects remain to be fully elucidated, the induction of innate immune memory appears to be the most plausible explanation. Understanding the pathophysiology of the disease in detail will eventually lead to better designs of novel trained immunity approaches. An important question to be addressed is to reveal the pathway that disseminates a state of ‘trained immunity’ and protection against SARS-CoV-2 infection and its variants of concern. More detailed investigations in this direction may lead to plausible answers to whether BCG can impart protective long-lasting immunity against SARS-CoV-2 and other respiratory infections.

## Figures and Tables

**Table 1:** Studies on the non-specific effects of BCG vaccination on various infections.

Cross-Protection	Model	Remarks
*L. major*	Mice	30–50% [1]
*C. albicans*	Mice	100% survival in BCG-vaccinated mice vs. 30% in control mice [2]
Influenza A	Mice	100% of mice immunized intranasally with BCG survived lethal IAV infection [3]
HSV1	Mice	BCG-inoculated mice: 41%; survival in control: 18% [4]
Pneumonia	Children (case-controlled study)	50% protection [5]
Sepsis	Case–control study	Reduced childhood mortality; better long-term survival [6]
Cutaneous malignant melanoma	Case–cohort study	No strong beneficial effect [7]
Pneumonia and sepsis	Randomized controlled trials	43% reduction in infectious disease mortality; 38% reduction within neonatal period [8]
Respiratory tract infections	Data analysis based on Demographic and Health Surveys data	17% to 37% risk in reduction [9]
Elderly pneumonia	Clinical trials	The risk of pneumonia was significantly decreased [10]
Acute URTIs respiratory tract infection	Humans	Protective effect on URTIs [11]
Acute lower respiratory tract infection (ALRI)	Infants	BCG vaccination may have a non-targeted protective effect against ALRI [12]
Leishmania amazonensis	Humans	A strong association between the increase of the frequency of innate immune system cells and the healing of lesions [13]
Influenza virus	Human study	Combined vaccination of BCG and influenza improved immunity against pandemic influenza A (H1N1) [14]
SARS-CoV-2	Healthy elderly individuals	BCG vaccination down-regulates circulating inflammatory markers IL-10 and IL-33 and does not lead to increased inflammation in elderly individuals [15]

**Table 2:** Clinical trials using BCG vaccine against SARS-CoV-2.

S. No	Clinical Trial Number	Location	Title	Date of Recruitment	Interventions
1	NCT04328441	Netherlands	Reducing Health Care Workers’ Absenteeism in COVID-19 Pandemic Through BCG Vaccine (BCG-CORONA)	31 March 2020	BCG vaccine vs. placebo
2	NCT04659941	Brazil	Use of BCG Vaccine as a Preventive Measure for COVID-19 in Health Care Workers (ProBCG)	9 December 2020	BCG Vaccine
3	NCT04347876	Egypt	Outcome of COVID-19 Cases Based on Tuberculin Test: Can Previous BCG Alter the Prognosis?	15 April 2020	Diagnostic Test: Tuberculin test
4	NCT04348370	USA	BCG Vaccine for Health Care Workers as Defense Against COVID-19 (BADAS)	16 April 2020	BCG vaccine vs. placebo
5	NCT04350931	Egypt	Application of BCG Vaccine for Immune Prophylaxis Among Egyptian Healthcare Workers During the Pandemic of COVID-19	17 April 2020	BCG vaccine vs. placebo
6	NCT04362124	Columbia	Performance Evaluation of BCG Vaccination in Healthcare Personnel to Reduce the Severity of SARS-CoV-2 Infection	24 April 2020	BCG vaccine vs. placebo
7	NCT04369794	Brazil	COVID-19: BCG As Therapeutic Vaccine, Transmission Limitation, and Immunoglobulin Enhancement (BATTLE)	30 April 2020	BCG vaccine vs. placebo
8	NCT04373291	Denmark	Using BCG Vaccine to Protect Health Care Workers in the COVID-19 Pandemic	4 May 2020	BCG-Denmark vs. saline
9	NCT04379336	South Africa	BCG Vaccination for Healthcare Workers in COVID-19 Pandemic	7 May 2020	BCG vaccine vs. placebo
10	NCT04384549	France	Efficacy of BCG Vaccination in the Prevention of COVID19 Via the Strengthening of Innate Immunity in Health Care Workers (COVID-BCG)	12 May 2020	BCG vaccine vs. placebo
11	NCT04414267	Netherlands	Bacillus Calmette-Guerin Vaccination to Prevent COVID-19 (ACTIVATEII)	4 June 2020	BCG vaccine vs. placebo
12	NCT04461379	Mexico	Prevention, Efficacy and Safety of BCG Vaccine in COVID-19 Among Healthcare Workers	8 July 2020	BCG vaccine vs. placebo
13	NCT04475302	India	BCG Vaccine in Reducing Morbidity and Mortality in Elderly Individuals in COVID-19 Hotspots	17 July 2020	BCG vaccine (Freeze-dried)
14	NCT04534803	USA	BCG Against COVID-19 for Prevention and Amelioration of Severity Trial (BAC to the PAST)	1 September 2020	BCG vaccine vs. placebo
15	NCT04537663	Netherlands	Prevention of Respiratory Tract Infection And COVID-19 Through BCG Vaccination in Vulnerable Older Adults (BCGPRIME)	3 September 2020	BCG vaccine vs. placebo
16	NCT04542330	Denmark	Using BCG to Protect Senior Citizens During the COVID-19 Pandemic	9 September 2020	BCG-Denmark vs. saline
17	NCT04327206	Australia	Efficacy of BCG Vaccination in the Prevention of COVID19 Via the Strengthening of Innate Immunity in Health Care Workers (BRACE)	31 March 2020	BCG vaccine vs. 0.9% NaCl
18	NCT04632537	United States	BCG Vaccination to Prevent COVID-19 (NUEVA)	17 November 2020	Tice BCG vs. saline
19	NL8547	Netherlands	Reducing Hospital Admission of Elderly in Sars-Cov-2 Pandemic Via the Induction of Trained Immunity By Bacillus Calmette-Guerin Vaccination, A Randomized Controlled Trial (BCG-CORONA Elderly)	May 2020	BCG vs. placebo
20	CTRI/2020/05/025013	India	Evaluation of BCG as potential therapy for COVID-19	6 May 2020	BCG vaccine vs. saline
21	NCT04641858	Denmark	BCG to Reduce Absenteeism Among Health Care Workers During the COVID-19 Pandemic (EDCTP)	24 November 2020	BCG vaccine Danish strain vs. saline
22	IRCT202004 11047019N1	Iran	Investigating the Effect of BCG Vaccine on Preventing COVID-19 Infection in Healthcare Staff Exposed to SARS-CoV-2	May 2020	BCG vaccine vs. saline
23	EUCTR2020-001888- 90-DK	Denmark	To Reduce Absenteeism among Health Care Workers with Direct Patient Contact during the COVID-19 Pandemic	30 April 2020	BCG Danish strain vs. Placebo
24	EUCTR2020-002503- 19-GB	Spain; Australia; Netherlands; United Kingdom	BCG Vaccination to Reduce the Impact of COVID-19 on Health Care Workers	8 July 2020	BCG vs. Placebo
25	CTRI/2020/04/024833	India	Effect of BCG-Denmark (Green Signal) on Prevention of COVID-19 Infection in Health Care Workers—A Double-Blind Randomized Controlled trial	1 May 2020	BCG vs. saline

## Data Availability

Not applicable.

## Abbreviations

NTM: non-tuberculous mycobacteria, PAMPs: pathogen-associated molecular patterns, TIbVs: trained immunity-based vaccines, AIR: adaptive immune response, PRR: pattern recognition receptor, APCs: antigen-presenting cells, HSCs: hematopoietic stem cells, AIR: adaptive immune response, IIR: innate immune response, OPV: oral polio vaccine, ADCC: antibody-dependent cellular cytotoxicity, AICs: adaptive immunity cells, ACE-2: angiotensin-converting enzyme 2, RBD: receptor-binding domain.
